# Diverse roles of UBE2S in cancer and therapy resistance: Biological functions and mechanisms

**DOI:** 10.1016/j.heliyon.2024.e24465

**Published:** 2024-01-18

**Authors:** Mengjun Zhang, Jialin Wang, Zidi Zhang, Yan Guo, Xueling Lou, Lindong Zhang

**Affiliations:** a Department of Gynecology, The Third Affiliated Hospital of Zhengzhou University, 7 Rehabilitation Front Street, Zhengzhou 450052, China; b Department of Orthopedics, Xuanwu Hospital, Capital Medical University, Beijing, 100000, China; c Department of Oncology, Henan Provincial People's Hospital, People's Hospital of Zhengzhou University, No. 7 Weiwu Street, Zhengzhou 450003, China

**Keywords:** UBE2S, Pan-cancer, Therapy resistance, Biological function, Mechanism

## Abstract

The Ubiquitin Conjugating Enzyme E2 S (UBE2S), was initially identified as a crucial member in controlling substrate ubiquitination during the late promotion of the complex's function. In recent years, UBE2S has emerged as a significant epigenetic modification in various diseases, including myocardial ischemia, viral hepatitis, and notably, cancer. Mounting evidence suggests that UBE2S plays a pivotal role in several human malignancies including breast cancer, lung cancer, hepatocellular carcinoma and etc. However, a comprehensive review of UBE2S in human tumor research remains absent. Therefore, this paper aims to fill this gap. This review provides a comprehensive analysis of the structural characteristics of UBE2S and its potential utility as a biomarker in diverse cancer types. Additionally, the role of UBE2S in conferring resistance to tumor treatment is examined. The findings suggest that UBE2S holds promise as a diagnostic and therapeutic target in multiple malignancies, thereby offering novel avenues for cancer therapy.

## 1 Introduction

The process of ubiquitination, which involves the post-translational modification of target proteins by the peptide ubiquitin's covalent attachment, was first discovered in the early 1980s. The process is carried out by three enzymes: ubiquitin-ligase E3, ubiquitin-binding enzyme E2, and ubiquitin-activating enzyme E1. A crucial role is played by ubiquitin-binding enzyme E2 in regulating protein fate by binding to various types of E3. Currently, there are 38 E2 enzymes identified in humans, and some of them, such as Ube2Q2, UBE2S, Ube2T2, and UbcH13, have been linked to the malignant biological behavior of different tumors and may serve as potential therapeutic targets. The ubiquitin-binding enzyme E2S (UBE2S) is a significant E2 key enzyme that plays a crucial role in the APC-catalyzed polyubiquitination process. Recent research has indicated that UBE2S is closely linked to various aspects of human tumourigenesis, metastasis, prognosis, and therapeutic sensitivity. As a result, UBE2S has garnered significant attention as a risk factor in clinical and basic studies, particularly in oncology-related studies. Furthermore, UBE2S is highly expressed in a variety of malignancies, including brain, cervical, colon, liver, ovarian, lung, breast, and endometrial cancers.

UBE2S is implicated as a crucial role in the pathogenesis and progression of various malignancies, exerting its effects through promoting cell invasion, migration and proliferation, as well as inhibition of apoptosis and dysregulation of the cell cycle. Extensive basic research has elucidated the mechanism by which UBE2S elongates ubiquitin chains to specific substrate proteins and promotes degradation through 26 S proteasome binding to UBE2C. However, whether UBE2S can be used as a biomarker for cancer detection and a therapeutic target remains to be investigated, for example, as a diagnostic biomarker for pan-cancer? Which signaling pathways are involved in regulating cancer? Which downstream target genes are regulated? Are they involved in the therapeutic resistance of cancer? With a series of questions and hypotheses, this paper reviews the differential expression of all identified UBE2S involved in tumor progression, their corresponding biological functions and the molecular mechanisms involved in their regulation, with the aim of opening up new horizons for treatment and diagnosis of cancers. Ubiquitination is a fundamental process that significantly impacts various biological functions, such as protein degradation, signal transduction, and intracellular homeostasis. Emerging evidence strongly suggests that ubiquitination plays a pivotal role in the progression of cancer. The central role in ubiquitin signaling is held by the ubiquitin-binding enzymes (E2s), which govern crucial aspects of this cascade. Therefore, the precise regulation of these enzymes is imperative for maintaining intracellular homeostasis. Among these proteins, UBE2S, a k11 chain-specific E2 enzyme associated with the human APC/C and characterized by a 24 kDa peptide, assumes a pivotal function in both mitosis and meiosis. UBE2S is responsible for ensuring accurate ubiquitination of cell cycle regulators during mitosis. Nevertheless, the existing literature lacks comprehensive investigations concerning the involvement of UBE2S in various tumor types, encompassing its specificity, therapeutic response in diverse tumor microenvironments and immune microenvironments, as well as its diagnostic and therapeutic biological targets. Therefore, a systematic and detailed review of its structure, function, mechanism, and clinical application prospects is needed to facilitate the clarification of future research directions and clinical translation. The current research on UBE2S genes in various human tumors lacks comprehensive integration of their roles across different cancer types. This includes a dearth of summary analyses elucidating their functions as either oncogenes or tumor suppressors, as well as a lack of comprehensive summaries regarding their potential as diagnostic and prognostic biomarkers. Furthermore, there is a paucity of knowledge regarding the distinct mechanisms of action exhibited by UBE2S in different tumor types, and a lack of exploration into the mechanisms underlying its drug resistance. Hence, this review aims to serve as a crucial point of reference and guidance for future investigations and clinical application of UBE2S genes, by comprehensively elucidating their functional and mechanistic profiles across various tumor types, as well as their involvement in therapeutic resistance.

## 2 Methods

A comprehensive literature review was conducted to locate relevant research articles pertaining to studies on the UBE2S gene. All articles were sourced from the Web of Science and PubMed databases, with data collected up until May 14, 2023. To facilitate the search process, various combinations of phrases and keywords such as “UBE2S,” “cancer,” “ubiquitination,” “therapeutic resistance,” and “signaling pathway” were utilized. Inclusion and exclusion criteria were applied to determine the eligibility of articles for analysis. The inclusion and exclusion criteria were as follows: 1. Only full-text articles were deemed eligible for further analysis. 2. Duplicate publications and those not written in English were excluded. 3. Review articles, dissertations, book chapters, and studies lacking result validation were also excluded. 4. Articles with inadequate study design or inconsistent results were excluded from the search. 5. Both peer-reviewed articles and preprints were also excluded. A total of 119 articles were initially identified during the search process, out of which 45 articles met the inclusion criteria. The assessment of these articles was conducted by two authors, namely L. Z and J. W. In cases of disagreement, a third author, Y. G, was consulted to resolve any discrepancies.

## 3 Structure and function of UBE2S

**Fig. 1:**
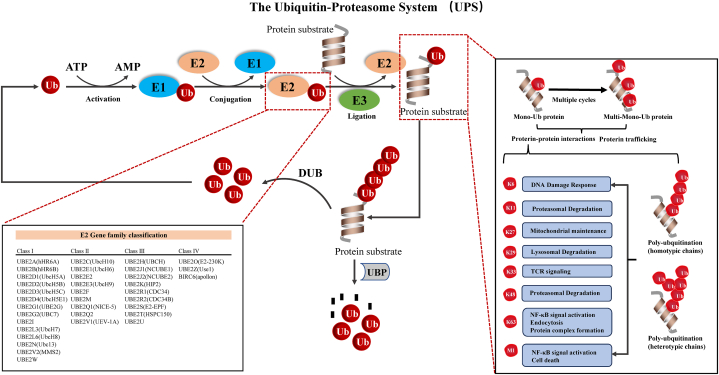
Schematic diagram of the ubiquitination process and a taxonomic map of the E2 gene family.

The ubiquitin modification process encompasses three primary layouts, namely single ubiquitination, homotypic polyubiquitination, and heterotypic polyubiquitination, as depicted in schematic diagram 3^1^. Post-translational modifications are ubiquitous in cellular proteins throughout their life cycle [1]. Any aberrations in ubiquitination modifications may have significant impacts on protein stability, cellular localization, and biological activity (Fig. 1) [2].

Within this system, a pivotal role of the ubiquitin-binding enzyme (E2) is to ensure that ubiquitin is transported from the ubiquitin-activating enzyme (E1) to the substrate and the ubiquitin-ligase (E3). The ubiquitin-binding enzymes are classified into four major types, with over thirty enzymes identified. E2 enzymes are categorized based on the existence or non-existence of supplementary extensions to their catalytic core. Class I E2s solely comprise the catalytic structural domain, while class III and II E2s additionally possess either the C-terminus or the N-terminus, and Class IV E2s possess both. These extensions result in distinct functional variances among E2s, such as variations in subcellular localization, stability of interaction with E1 enzymes, and activity of interaction with E3 enzymes (Fig. 1) [3].

**Fig. 2:**
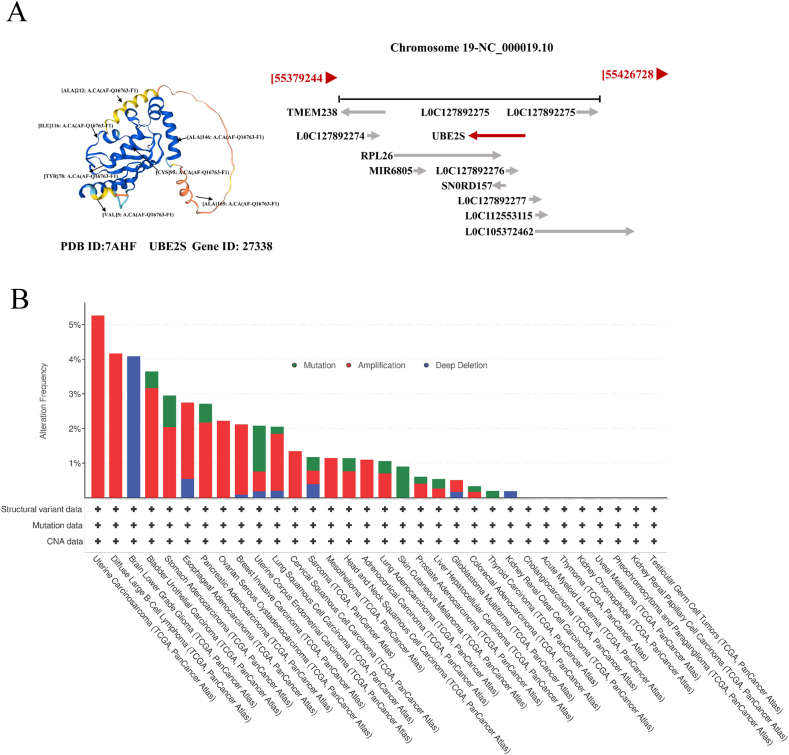
Structure and mutation of the UBE2S gene. (A) Schematic diagram of the three-dimensional structure and gene sequence. (B) Mutation characteristics of UBE2S in different tumors of TCGA were analyzed using the cBioPortal tool. The three types of mutations (green: Mutation, red: Amplification, blue: Deep Deletion) and the frequency of mutation site changes are shown in the figure. (For interpretation of the references to color in this figure legend, the reader is referred to the Web version of this article.)

The protein UBE2S, also referred to as E2EPF, is a decoded component of the E2s. The 3D structure and sequence structure schematic of UBE2S were obtained from the National Center for Biotechnology Information (NCBI, https://www.ncbi.nlm.nih.gov/) and the Human Protein Atlas (HPA, https://www.proteinatlas.org), as depicted in Fig. 2A–B. This protein is predominantly localized in the in the cytoplasm and nucleoplasm. UBE2S facilitates the transfer of ubiquitin from the E1 complex and facilitates its covalent attachment to various proteins [4]. A pivotal role is played by UBE2S in regulating the cell cycle and controlling mitotic by elongating the degradation of APC/C substrates through polyubiquitination-enhanced proteasomes catalyzed by the EBE2C/UBCH10 enzyme “Lys-11” linkage [[5], [6], [7]]. Additionally, UBE2S is involved in the ubiquitination degradation of the ubiquitin chain initiated by UBE2D1/UBCH5 and VHL (Von Hippel-Lindau), leading to the accumulation of HIF1A (HIF-1 alpha). Ubiquitination is not only involved in the regulation of protein quantity, but also involves different ubiquitination chain lengths (mono-ubiquitination, poly-ubiquitination, and poly-ubiquitination) and various ubiquitination chain types (connected through Met 1, Lys 6, Lys 11, Lys 27, Lys 29, Lys 33, Lys 48 and Lys 63) play extremely important regulatory functions in protein activity, protein-protein interactions and protein subcellular localization (Fig. 1). In vitro, UBE2S is capable of promoting polyubiquitination using all seven ubiquitin Lys residues [[8], [9], [10]]. K48 and K29-linked polyubiquitin chains, which serve as classically important members of endoplasmic reticulum associated degradation (ERAD) and 26 S proteasomal degradation [11]. In addition, K63-linked polyubiquitin chains facilitate various cellular processes such as protein translation, classification, complex formation, phosphorylation, transcription, autophagy, endocytosis, DNA repair, and RNA splicing [[12], [13], [14], [15], [16], [17], [18], [19], [20]]. UBE2S, a K11-specific strand extension E2 of APC/C, is reliant on strand initiation of UbcH10 and promotes the degradation of mitotic regulators. Due to the diversity and multivalency of UBE2S involved in ubiquitination, it may be widely involved in various physiological processes, including cell proliferation, apoptosis, autophagy, endocytosis, DNA damage repair, and immune response.

Mutations in genes involved in the ubiquitination or deubiquitination pathways, or dysregulation of the ubiquitin system, have been linked to a diverse range of human diseases, including but not limited to various cancers, neurodegenerative disorders, and metabolic abnormalities [21,22]. The study revealed that UBE2S is upregulated in certain types of cancer and acts as an oncogene, as evidenced by Fig. 4 and Table 1. Its primary role in pan-cancer involves the regulation of cell cycle, malignant cell proliferation, and apoptosis. Specifically, UBE2S facilitates the transfer of ubiquitin from the E1 complex and catalyzes its covalent attachment to other proteins, leading to “Lys-11” chain polyubiquitination. This process subsequently promotes the formation of the cell cycle-regulated ubiquitin ligase complex/loop (APC/C) with E3 ligase, which governs the malignant progression of cells during mitosis [[5], [6], [7]].

**Table 1:** Summary of UBE2S studies in tumor.

Cancer Type	Role	Mechanism
Lung cancer	Oncogene	UBE2S regulates Wnt/β-catenin signaling and promotes the progression
Lung cancer	Oncogene	UBE2S promotes the proliferation and survival through the P53 signal pathway
Lung cancer	Oncogene	UBE2S activates NF-κB signaling by binding with IκBα and promotes metastasis of lung adenocarcinoma cells
Endometrial cancer	Oncogene	UBE2S mediates tumor progression via SOX6/β-Catenin signaling
Liver cancer	Oncogene	UBE2S enhances the ubiquitination of p53 and exerts oncogenic activities
Liver cancer	Oncogene	UBE2S expression is elevated in hepatocellular carcinoma and predicts poor prognosis
Liver cancer	Oncogene	UBE2S promotes pancreatic cancer progression through VHL/HIF-1α/STAT3 signaling
Liver cancer	Oncogene	UBE2S was significantly associated with decreased overall survival and disease-free survival in HCC patients.
Pancreatic cancer	Oncogene	UBE2S stabilizes β-Catenin through K11-linked polyubiquitination to promote colorectal cancer development
Colorectal cancer	Oncogene	UBE2S promotes the invasion and metastasis of breast cancer and other malignant phenotypes.
Breast cancer	Oncogene	UBE2S promotes P21 degradation to promote the proliferation of oral cancer
Oral cancer	Oncogene	UBE2S promotes the progression and Olaparib resistance of ovarian cancer through Wnt/β-catenin signaling pathway
Ovarian cancer	Oncogene	The UBE2S/TSC1/mTOR axis mediates the mTOR pathway and promotes the progression of bladder cancer
Bladder Cancer	Oncogene	UBE2S is regulated by the PTEN/Akt pathway leading to chemotherapy resistance in glioblastoma
Glioma	Oncogene	UBE2S may contribute to the HIF-1 signal of cervical cancer
Glioma	Oncogene	UBE2S expression strongly correlates with glioma malignancy and resistance to chemo-radiotherapy.
cervical cancer	Oncogene	UBE2S interacts with TRIM28 to regulate cell cycle and promote hepatocellular carcinoma progression
melanoma	Oncogene	UBE2S is involved in protein degradation and signal transduction leading to the progression of melanoma

**Fig. 3:**
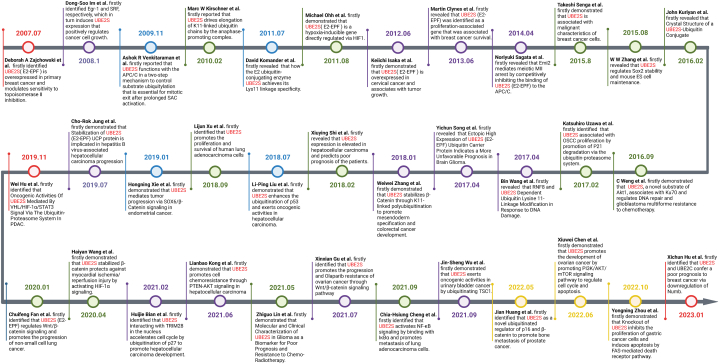
Timeline of UBE2S studies in tumors. This figure briefly shows the important studies and time points in the process of exploring the UBE2S gene.

Aside from regulating the cell cycle, the overexpression of UBE2S has been found to target the degradation of p53 tumor suppressor to promote tumor cell invasion, migration and proliferation [23]. Furthermore, the inhibition of ubiquitination and degradation of p53 in gastric cancer cells through knockdown of UBE2S may lead to the activation of endogenous FAS-mediated apoptotic pathway [24]. Moreover, aside from its impact on the malignant phenotype of tumors, the suppression of UBE2S impeded the repair of double-strand breaks (DSBs) facilitated by non-homologous end-joining (NHEJ), thereby enhancing the susceptibility of chemotherapy [25,26]. Therefore, UBE2S is frequently regarded as an oncogenic factor and is intimately linked to resistance to tumor therapy, and the targeting of UBE2S may represent a promising therapeutic approach. Fig. 3 showed the important time nodes of research on UBE2S.

**Fig. 4:**
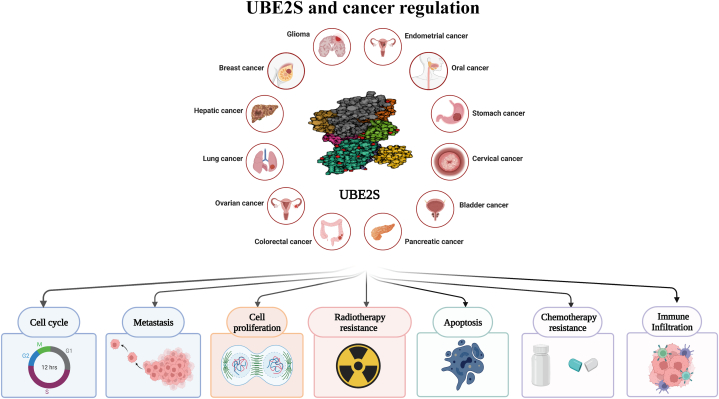
The mechanisms by which UBE2S mediates tumor regulation in pan-cancer include regulation of cell cycle, metastasis, cell proliferation, apoptosis, immune infiltration, and radiotherapy resistance.

## 4 Expression and function of UBE2S in cancer

### 4.1 Differential expression of UBE2S in different cancer

**Fig. 5:**
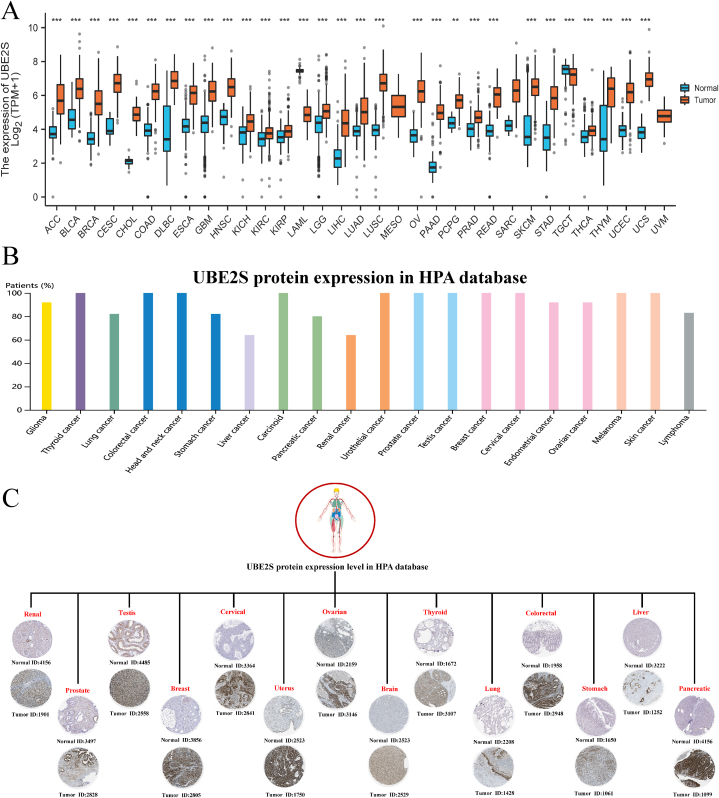
Differential expression of UBE2S genes in pan-cancerous tissues. (A) Transcript expression of UBE2S genes in 33 human normal and tumor tissues obtained from TCGA and GETx databases. Blue color indicates the expression level of the gene in normal tissues and red color indicates the expression level of the gene in tumor tissues. *P < 0.05, **P < 0.01, ***P < 0.001. (B) Statistical histogram of protein expression of the UBE2S gene in pan-cancerous tissues from the HPA database. (C) Immunohistochemical staining plots of the UBE2S gene in normal and tumor tissues in the HPA database in pan-cancer. The intensity of gene protein expression was determined based on staining intensity. (For interpretation of the references to color in this figure legend, the reader is referred to the Web version of this article.)

**Fig. 6:**
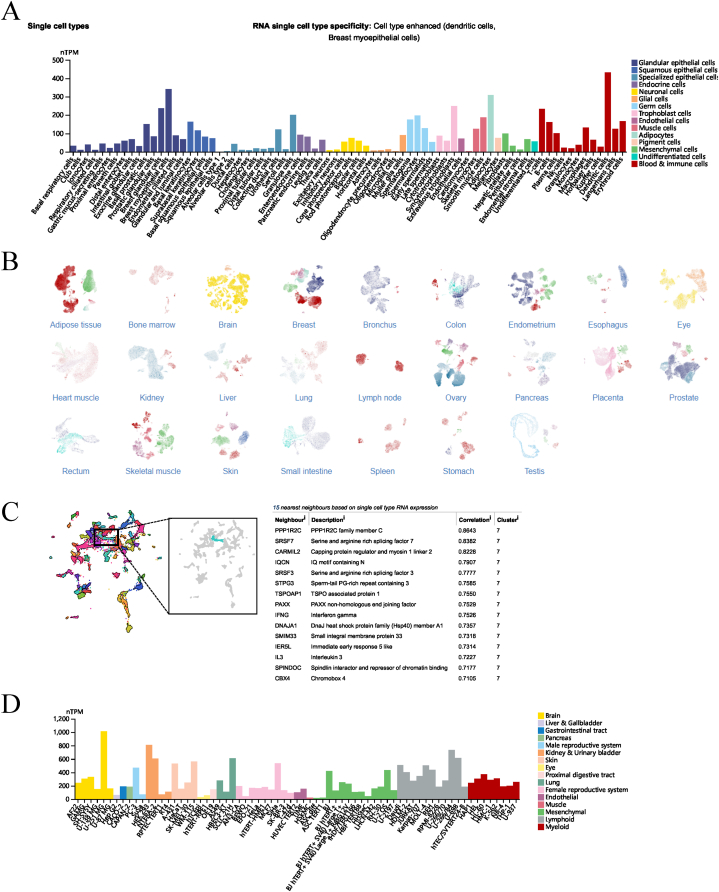
Differential expression of UBE2S gene in human single cells. (A) Histogram of transcript expression of UBE2S gene in single cells analyzed in different human organs. (B) Single-cell component profiles of transcriptional expression of UBE2S gene in single cells analyzed in different human organs. (C) Single-cell analysis of the top 15 genes most associated with the expression of UBE2S gene expression. (D) Differential expression of UBE2S genes in different cell lines in different human organs. Histogram of expression levels in tumor cells and normal cell lines.

In this study, the expression level of UBE2S gene in various cancers, that is, pan-cancer, was evaluated by nucleic acid level, protein level, and cell level analysis (Fig. 5). To this end, a dataset comprising 15,776 patients with various types of cancer from the GTEx (Genotype-Tissue Expression) and TCGA (The Cancer Genome Atlas Program) databases, encompassing 33 human malignant tumors and their corresponding normal controls, was utilized. The results, as depicted in Fig. 5A, revealed that the UBE2S gene was aberrantly expressed in 30 out of 33 types of cancers. Notably, Fig. 5A demonstrated that compared with corresponding normal tissues, the expression levels of UBE2S were significantly higher in 28 types of tumor tissues (UCS, UCEC, THYM, THCA, STAD, SKCM, SARC, READ, PRAD, PCPG, PAAD, OV, LUSC, LUAD, LIHC, LGG, KIRP, KIRC, KICH, HNSC, GBM, ESCA, DLBC, COAD, CHOL, CESC, BRCA, BLCA, and ACC). The protein expression level of UBE2S at the pan-cancer level was investigated with the help of the HPA (Human Protein Atlas) database. The results depicted in Fig. 5B–C indicate that UBE2S exhibited significantly elevated expression in immunohistochemical staining across various tumors. Specifically, immunohistochemical staining was conducted to assess UBE2S expression levels in 14 different types of tumors, including glioma, thymic adenocarcinoma, lung cancer, colorectal cancer, gastric cancer, ovarian cancer, endometrial cancer, cervical cancer, breast cancer, testicular cancer, prostate cancer, urothelial cancer, kidney cancer, pancreatic cancer, and liver cancer. The HPA database's single cell analysis depicted in Fig. 6A–D reveals that UBE2S exhibits anomalous expression in malignant cell lines. Moreover, the high expression cluster of the top 15 proteins (PPP1R2C, SRSF7, CARMIL2, IQCN, SRSF3, STPG3, TSPOAP1, PAXX, IFNG, DNAJA1, SMIM33, IER5L, IL3, SPINDOC, CBX4) has been demonstrated to impact the apoptosis, cell cycle, invasion, migration and proliferation of various human tumor cells. In summary, our analysis of public databases revealed that UBE2S exhibits frequent aberrant expression in human pan carcinomas. This finding is consistent with a substantial body of prior research indicating the significance of the UBE2S gene in the development of malignant phenotypes across various types of cancer. Consequently, there is a pressing need to investigate the potential prognostic significance of UBE2S in pan-cancer patients, and to elucidate the underlying molecular mechanisms governing its biological function and regulation.

### 4.2 Effect of UBE2S on the prognosis of cancer patients

**Fig. 7:**
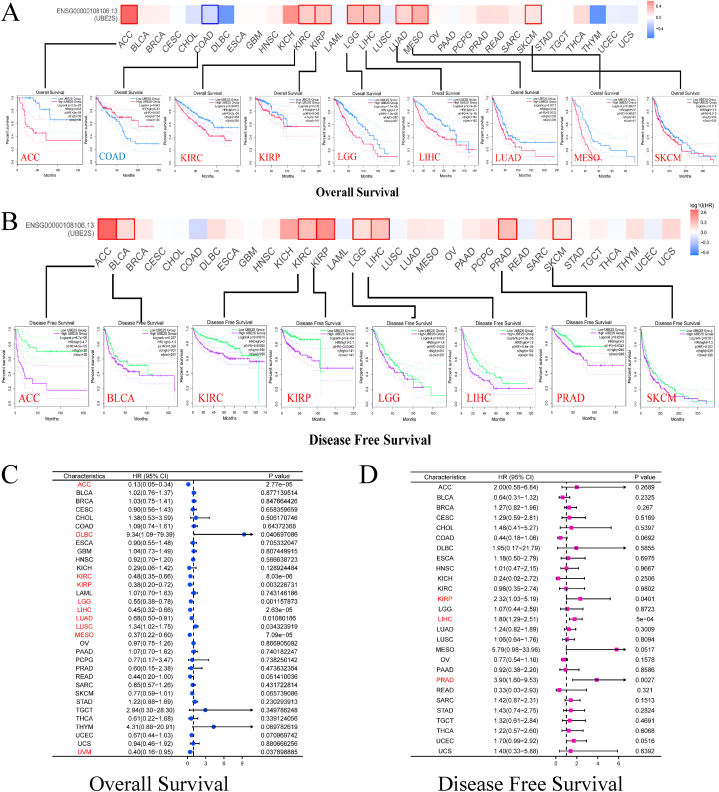
Association of UBE2S gene expression with survival and prognosis of multiple tumors in TCGA. (A–B) We analyzed the correlation between UBE2S gene expression and overall and disease-free survival in different tumors in TCGA using the GEPIA2 tool (survival heat map and Kaplan-Meier curve). (C–D) Forest plots of UBE2S gene expression in 33 different types of human tumors for univariate analysis of overall survival and disease-free survival, with visible HR intervals and P values, and statistically significant tumor types in univariate analysis of UBE2S in red. (For interpretation of the references to color in this figure legend, the reader is referred to the Web version of this article.)

What is the prognostic value of the UBE2S gene in patients with pan-cancer? The present study evaluated the prognostic significance of UBE2S by analyzing expression variations and patient survival data obtained from the TCGA and GEPIA databases. The results depicted in Fig. 7A indicate that patients with higher expression levels of UBE2S had significantly lower overall survival in SKCM, MESO, LUAD, LIHC, LGG, KIRP, KIRC, and ACC. Similarly, Fig. 7B demonstrates a significant association between high UBE2S expression and shorter disease-free survival in patients with SKCM, PRAD, LIHC, LGG, KIRP, KIRC, BLCA, and ACC. Furthermore, the risk factor analysis for OS and DFS was performed at the pan-cancer level to explore the impact of UBE2S on prognosis, as depicted in Fig. 7C–D. Notably, aberrantly elevated UBE2S expression was identified as a significant determinant of overall survival in patients with UVM, MESO, LUSC, LUAD, LIHC, LGG, KIRP, KIRC, DLBC, and ACC (P < 0.05). Moreover, UBE2S was found to exert an impact on disease-free survival in KIRP, LIHC, and PRAD tumors (P < 0.05). In summary, our study has revealed a significant association between the overexpression of UBE2S in various types of cancer, including neurological, urological, gastrointestinal, and gynecological genital tumors, and unfavorable patient prognosis. Consequently, a comprehensive understanding of the precise impact of UBE2S on patient survival and potential oncogenic pathways is imperative.

### 4.3 Functions of UBE2S in cancer

**Fig. 8:**
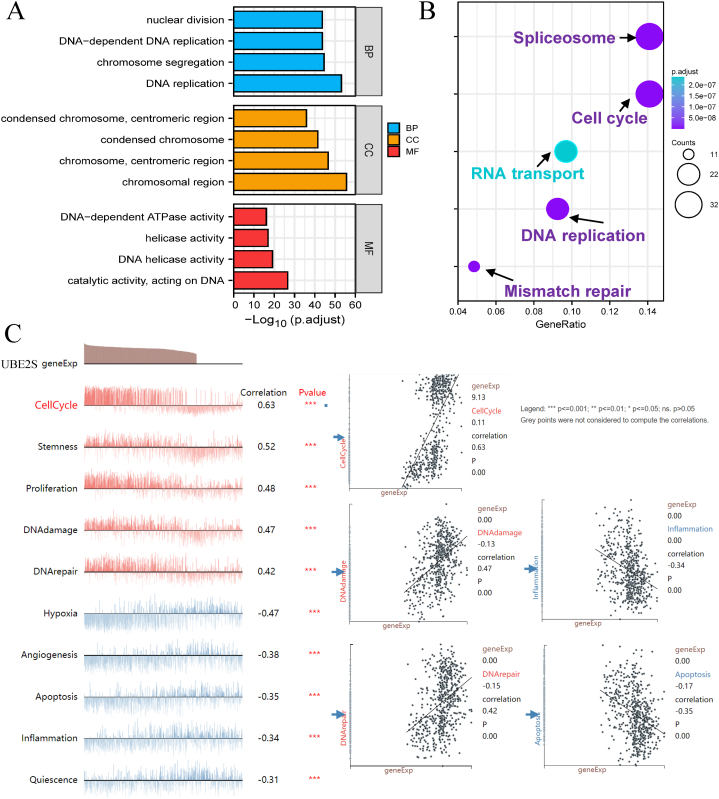
Go (Gene Ontology) functional annotation and KEGG (Kyoto Encyclopedia of Genes and Genomes) pathway enrichment analysis of UBE2S in pan-cancer. (A) Functional annotation, cellular component, molecular function (B) KEGG pathway enrichment analysis (Splicepsome, Cell cycle, RNA transport, DNA replication, Mismatch repair). (C) GSEA database enrichment analysis results for UBE2S (Cell cycle signaling pathways, proliferation, DNA damage, angiogenesis, apoptosis, inflammatory signaling pathways, etc.). A scatter plot of correlations with statistically significant differences in 5 correlations (cell cycle, DNA damage, inflammation, DNA repair, and apoptosis) is visible in the figure.

The present study utilized GO (Gene Ontology) and KEGG (Kyoto Encyclopedia of Genes and Genomes) analyses to study the possible biological functions and corresponding molecular mechanisms of UBE2S at the pan-cancer level. The results of the GO functional annotation analysis are presented in Fig. 8A and Table 2, revealing enriched biological processes such as nuclear division and DNA replication. Moreover, the results of KEGG demonstrated that the primary enriched signaling pathways include cell cycle, spliceosome, DNA replication, RNA transport, and mismatch repair, as depicted in Fig. 8B and Table 3. Significantly, our findings indicate a close association between the plentiful functions and signaling pathways of UBE2S in pan-cancer and the regulation of cell cycle and repair of DNA damage, which are of fundamental significance in numerous human tumors. To corroborate the degree of enrichment of UBE2S in pan-cancer signaling pathways, we utilized the results of GSEA analysis to demonstrate that the most crucial enriched signaling pathways (P < 0.05) were the DNA repair signaling pathway, DNA damage pathway, cell cycle signaling pathway, inflammation, and apoptosis (Fig. 8C)·In summary, the impact of UBE2S on the DNA repair, DNA damage, and the cell cycle may potentially govern the progression of malignancy and unfavorable survival consequences in individuals diagnosed with pancreatic cancer.

**Table 2:** Functional enrichment analysis of UBE2S in pan-cancer.

ONTOLOGY	ID	Description	Gene Ratio	Bg Ratio	P value
BP	GO:0006260	DNA replication	74/443	274/18,670	2.80e-57
BP	GO:0007059	chromosome segregation	71/443	321/18,670	2.19e-48
BP	GO:0006261	DNA-dependent DNA replication	53/443	153/18,670	2.04e-47
BP	GO:0000280	nuclear division	77/443	407/18,670	3.28e-47
BP	GO:0140,014	mitotic nuclear division	64/443	264/18,670	3.00e-46
CC	GO:0098,687	chromosomal region	82/457	349/19,717	5.76e-59
CC	GO:0000775	chromosome, centromeric region	59/457	193/19,717	1.25e-49
CC	GO:0000793	condensed chromosome	58/457	223/19,717	2.58e-44
CC	GO:0000779	condensed chromosome, centromeric region	42/457	118/19,717	1.17e-38
CC	GO:0000776	kinetochore	44/457	135/19,717	1.62e-38
MF	GO:0140,097	catalytic activity, acting on DNA	46/442	213/17,697	5.34e-30
MF	GO:0003678	DNA helicase activity	26/442	81/17,697	3.34e-22
MF	GO:0004386	helicase activity	32/442	163/17,697	7.75e-20
MF	GO:0008094	DNA-dependent ATPase activity	22/442	69/17,697	6.62e-19
MF	GO:0017,116	single-stranded DNA-dependent ATP-dependent DNA helicase activity	14/442	20/17,697	1.01e-18

**Table 3:** Enrichment analysis of signal pathway of UBE2S in pan-cancer.

ONTOLOGY	ID	Description	GeneRatio	BgRatio	pvalue
KEGG	hsa 03030	DNA replication	21/227	36/8076	4.04e-24
KEGG	hsa 04110	Cell cycle	32/227	124/8076	1.26e-22
KEGG	hsa 03040	Spliceosome	32/227	151/8076	8.43e-20
KEGG	hsa 03430	Mismatch repair	11/227	23/8076	6.83e-12
KEGG	hsa 03013	RNA transport	22/227	186/8076	9.60e-09

## 5 UBE2S-mediated signaling pathways in cancer

**Fig. 9:**
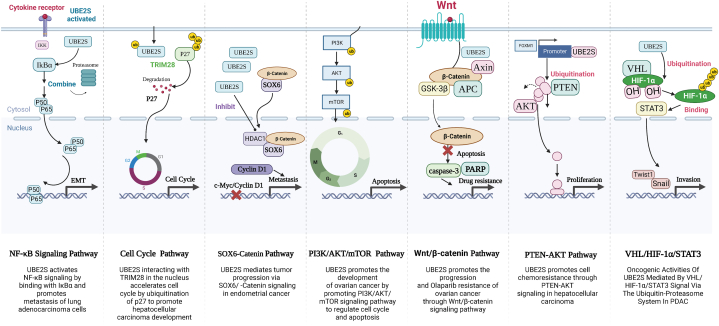
Schematic diagram of the different signaling pathway summary mechanisms of UBE2S in pan-cancer.

The burgeoning focus on ubiquitin-binding enzyme research has prompted investigations into UBE2S, revealing its distinctive biological functions and garnering significant interest in clinical research and medical advancement, particularly in the realm of oncology. Presently, mounting evidence suggests that UBE2S impacts cancer progression through multiple mechanisms, such as NF-κB, Cell cycle, SOX6-Catenin, Wnt/β-catenin, PI3K/AKT/mTOR, PTEN-AKT, and VHL/HIF-1α/STAT3 (Fig. 9).

### 5.1 NF-κB signaling pathway

A distinguishing feature of numerous types of cancer is the activation of the NF-κB signaling pathways. And anomalous NF-κB activation has been detected in a diverse range of solid tumors including cancers of the pancreas, breast, bladder, prostate, and lung [[27], [28], [29], [30]]. The ubiquitination-mediated regulation of several critical stages of this pathway is disrupted, thereby assuming a crucial coordinating function in cellular survival and death pathways, as well as in the immune system [[31], [32], [33]]. The activation of NF-κB results in the phosphorylation of I-κBα, which translocates from the cytoplasm to the nucleus, resulting in the degradation of IκB kinase (IKK). This process is implicated in the binding of promoter regions of target genes associated with proliferation, anti-apoptosis, and drug resistance [[34], [35], [36], [37]]. Notably, downstream targets of NF-κB, such as Snail and MMP-9, are involved in tumor cell metastasis through a specific signaling pathway called epithelial-mesenchymal transition (EMT) [[38], [39], [40], [41], [42], [43]]. An elevated level of UBE2S expression facilitates the migratory and invasive capabilities of cancer cells according to the findings of the study [42]. UBE2S activated the NF-κB signaling pathway in lung adenocarcinoma cells and triggered the expression of downstream EMT markers. Manipulation of UBE2S gene expression through knockdown or overexpression in adenocarcinoma cells resulted in increased nuclear translocation of p65 and heightened NF-κB activity. This activation may subsequently induce EMT signaling, which plays a crucial role in the ability of cancer cells to invade and migrate [[39], [40], [41], [42],^44^]. Furthermore, it was proposed that the administration of Ly294002 resulted in a reduction of UBE2S protein levels and an increase in IκBα protein levels within lung adenocarcinoma cells. Increased NF-κB activity and reduced IκBα expression have been found in various cancer. And it is a common signal transduction pathway to activate NF-κB by means of IKK to phosphorylate and degrade IκBα [45]. Furthermore, UBE2S is subject to stabilization via Akt phosphorylation, which serves to regulate the stability of the Ku70 complex that participates in DNA repair mechanisms [25]. Additionally, AKT has been documented to activate NF-κB signaling, which contributes to oncogenic transformation [46]. Ultimately, UBE2S facilitates the metastasis of lung adenocarcinoma cells by means of activating NF-κB signaling through its binding to IκBκ.

### 5.2 Cell cycle signaling pathway

Multiple phases of the cell cycle signaling pathway (including G1 phase, S phase and G2 phase) are commonly abnormal in a variety of tumors. The complex comprising Cyclin and CDK assumes a pivotal regulatory function in the cell cycle pathway. Prior research has suggested that UBE2S is a dedicated E2 enzyme of the multisubunit APC/C E3 enzyme that regulates cell cycle progression [47]. The E3 enzymes-associated cell cycle regulation is reliant on UBE2C for the elongation of polyubiquitin chains utilizing ubiquitin (Ub) and UBE2S as substrates. As such, a pivotal role is played by UBE2S in cell cycle regulation. The findings of the study indicate that in the nucleus, overexpression of TRIM28 and UBE2S facilitates p27 ubiquitination, thereby promoting the G1/S phase transition of hepatocellular carcinoma cells and ultimately leading to cell cycle progression [48].

### 5.3 SOX6-catenin signaling pathway

Ubiquitination is a reversible biochemical process that involves the binding of ubiquitin to substrate proteins, leading to proteins post-translational modification. This process takes part in various cellular functions and contributes to the development of colorectal carcinogenesis by stabilizing β-catenin through direct interaction and ubiquitinating their K19 residues via K11 linkage [49]. The activation of the protein pathway linked to β is a commonly observed phenomenon in tumors, which collaborates with the loss of PTEN to initiate and advance tumor growth [50]. Upon activation by FrizzledTM, Wnt signaling causes β-linked protein to dissociate from the degradation complex, which translocates to the nucleus where it associates with Tcf/Lef as a transcription complex. And a variety of downstream gene transcriptions that play a role in carcinogenesis are also initiated by this transcription complex [51]. Abnormally high expression of UBE2S promotes activation of β-linked protein signaling. Subsequent studies have shown that UBE2S could inhibit SOX6, which could regulate PD-L1 and Cyclin D1 upstream. UBE2S exerts its repressive effect on SOX6 at the transcriptional level [52]. Thus, it has been demonstrated that SOX6 could bind to β-catenin, disengaging it from the transcription complex and hindering its function in the nucleus. Nonetheless, abnormally high expression of UBE2S could hinder this critical process [52].

Furthermore, the transcriptional regulatory mechanism facilitated by UBE2S may contribute to the repression of SOX6 via UBE2S-mediated ubiquitination, which is subsequently followed by proteasome-dependent degradation [52]. Ultimately, the transactivation of endometrial cancer progression via SOX6/β-linked protein signaling is facilitated by UBE2S.

### 5.4 PI3K/AKT/mTOR signaling pathway

PI3K is a dimer composed of catalytic subunit p110 and regulatory subunit p85, which could bind to growth factor receptors including EGFR to regulate the protein structure of Akt. This modulation leads to the activation of Akt and the phosphorylation of downstream substrates, including apoptosis-associated proteins Bad and Caspase 9, thereby regulating various cellular phenotypes, including proliferation, differentiation, apoptosis, and migration [53]. Furthermore, Akt can activate IKK, which exhibits cross-talk with the NF-kB pathway. The PI3K/Akt pathway could be regulated downstream by mammalian target of rapamycin (mTOR), and this pathway is an intracellular signaling pathway that is activated by extracellular signals and promotes various biological processes, including angiogenesis, cell growth, cell proliferation and cell metabolism [54]. FoxO, c-Myc and HIF1α are all downstream transcription factors of mTOR. PI3K/AKT/mTOR pathway is mediated by the phosphorylation of downstream substrates at serine or threonine residues, with key genes involved being AKT/protein kinase b and phosphatidylinositol 3-kinase (PI3K). The transmission of PI3K-AKT signaling to downstream pathways results in the activation of various pathways, such as protein translation, cell cycle, apoptosis, and the P53 pathway [55]. The inhibition of PI3K/AKT/mTOR pathway by UBE2S could induce apoptosis and impede the cell cycle, thereby restraining the invasion and proliferation of ovarian cancer cells and correlating with poorer survival prognosis [56]. These findings imply that UBE2S holds potential as a molecular therapy and prognostic tool for ovarian cancer.

### 5.5 Wnt/β-catenin signaling pathway

An important factor leading to cancer is the Wnt/β-catenin signaling pathway, whose activation allows β-catenin to accumulate in the cytoplasm, eventually translocate to the nucleus to activate transcription factors. Additionally, there is empirical support indicating that Wnt signaling facilitates the aggregation of other transcriptional regulatory molecules associated with cancer, including TAZ and Snail 1, within the nucleus [57]. The classical Wnt/β-catenin signaling pathway involves the activation of target gene expression in the nucleus through the participation of various components, including APC, Axin, β-catenin, glycogen synthase kinase 3 (GSK3), Frizzled family transmembrane receptor protein Dishevelled, and TCF/LEF family transcriptional regulators, as well as Wnt family secreted proteins [51]. β-catenin functions as an essential E-calmodulin that serves as both a transcriptional co-regulatory molecule and an intercellular adhesion junction protein [58]. The APC/Axin/GSK-3β complex and CK1 is able to regulate β-catenin through coordinated phosphorylation and allow it to be ubiquitinated and degraded by the proteasome. The overexpression of UBE2S in cancer cells has been shown to significantly increase the β-catenin expression, including MMP7 and cyclin D1 proteins [59]. The modification of β-catenin by UBE2S resulted in increased stability and accumulation within cancer cells, ultimately promoting cancer progression. The study provided evidence of UBE2S-mediated upregulation of the Wnt/β-catenin pathway and downstream molecule expression in A549 cells [59].

### 5.6 PTEN-AKT signaling pathway

The investigation of tumor suppressors holds significant value in the therapeutic approach towards human cancers. Phosphatase and tensin homologs (PTEN) are tumor suppressor genes that possess dual specificity for lipid phosphatases and protein phosphatases, thereby governing numerous biological processes such as proliferation, survival, cell structure, motility, energy metabolism, and genomic stability [60]. Additionally, PTEN plays a crucial role in regulating multiple intracellular signaling pathways. The ubiquitination/deubiquitination mechanism is a major regulatory process that influences PTEN's stability and subcellular localization [61]. The transcription factor FOXM1 induces the upregulation of UBE2S in hepatocellular carcinoma cells, leading to the ubiquitination of PTEN at Lys327, Lys 60 and the phosphorylation of AKT, which enhances chemoresistance [62]. This effect can be mitigated by the metastable AKT inhibitor MK2206. The malignant phenotype of hepatocellular carcinoma could be promoted by the FOXM1/UBE2S/PTEN/p/AKT axis, indicating that it may be possible to treat this disease by targeting UBE2S [62].

### 5.7 VHL/HIF-1α/STAT3 signaling pathway

HIF1 is a transcription factor possessing a helix-loop-helix structure, which facilitates the activation of genes encoding proteins involved in the hypoxic homeostatic response. Initially identified as a DNA-binding protein responsible for mediating interferon-dependent gene expression, Signal transducer and activator of transcription (STAT) plays a pivotal role in this process. Numerous studies have substantiated that STAT3 undergoes hyperactivation in the majority of human cancers, thereby regulating a multitude of genes linked to the immune evasion, drug resistance, metastasis, invasion, angiogenesis, proliferation, and survival of cancer cells. These outcomes are frequently associated with unfavorable clinical prognoses. UBE2S is a cell cycle-regulated ubiquitin ligase and a crucial constituent of the anaphase-promoting complex/cyclosome (APC/C), which regulates the progression of cell mitosis. Consequently, UBE2S manages the degradation of the 26 S proteasome via the proteasome and facilitates mitotic exit, thereby serving as a pivotal factor in cell division progression [63]. The study revealed that in pancreatic cancer cells, the expression of the UBE2S gene facilitated the process of epithelial-mesenchymal transition (EMT) by means of the ubiquitin proteasome system and the interaction between UBE2S and VHL. This indicates UBE2S takes part in promoting endodermal metastasis and metastasis in pancreatic cancer cells in vitro and in vivo by inhibiting promoter activity in the VHL/HIF-1α/STAT3 pathway [63]. The upregulation of UBE2S through the standard ubiquitination response further promotes the metastasis and invasion of pancreatic ductal adenocarcinoma (PDAC) cells. Additionally, UBE2S may regulate the JAK2-STAT3 pathway through VHL, thereby promoting the proliferation of PDAC cells and facilitating the EMT process [63].

## 6 UBE2S in cancers

**Fig. 10:**
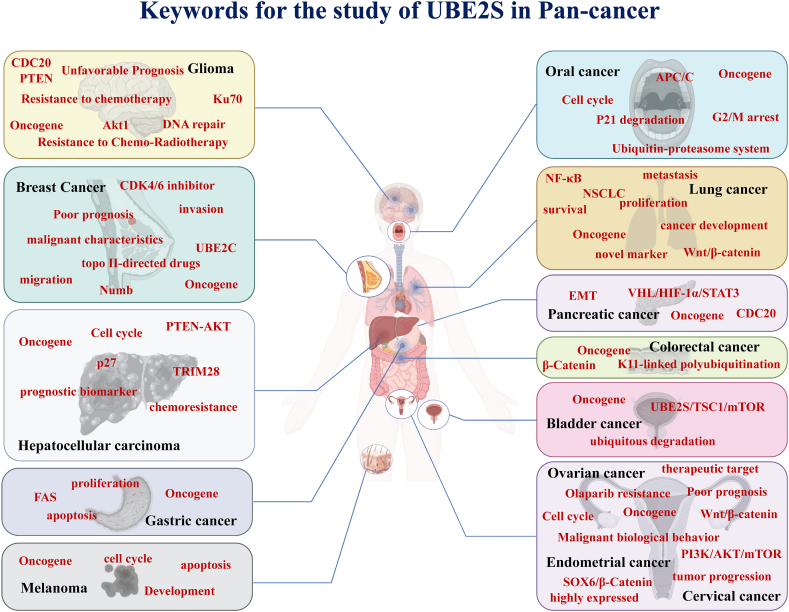
Schematic diagram of the keyword summary for the study of UBE2S in different human tumors.

Numerous studies have demonstrated that the occurrence and development of diverse types of malignant tumors could be affected by UBE2S (Fig. 10). Subsequently, we provide a concise overview of the investigations pertaining to the involvement of UBE2S in human cancers, organized by system, as depicted in Table 1.

### 6.1 The role of UBE2S in the female reproductive system

This research demonstrates the effects of UBE2S in 3 important cancers of the female reproductive system (cervical, endometrial, and ovarian). Our findings suggest that the downregulation of UBE2S expression by lignocaine and quercetin may inhibit epithelial-mesenchymal transition (EMT) and impede cervical cancer metastasis, thereby highlighting UBE2S as a promising therapeutic target for cervical cancer [43]. Furthermore, the impact of UBE2S on the ability of cell tumorigenesis, invasion and proliferation for the cervical cancer cell lines was observed to be mediated by the promotion of HIF-1α expression. Given UBE2S's involvement in mitotic surveillance, spindle assembly, and cell cycle regulation, an inquiry was made into the potential involvement of UBE2S in the cellular response to checkpoint drugs. The present study confirms that there is a close relationship between the abnormally high expression of UBE2S and chemoresistance to topotecan, etoposide, and doxorubicin. According to these researchs, UBE2S could have a significant impact on the growth and invasiveness of cervical tumor cells and may be closely linked to chemoresistance [64,65]. The upregulation of UBE2S in endometrial cancer has been demonstrated to be significantly associated with a poor prognosis. This upregulation exerts oncogenic activity through translocating the β-linked protein to the nucleus, followed by upregulation of cyclin D1 and c-Myc. Therefore, UBE2S could be a potential oncogene and prognostic risk factor, activating the UBE2S/SOX6/β-linked protein axis to function as an oncogene in the endometrium [52].

Research has demonstrated that UBE2S is significantly upregulated in ovarian cancer and is linked to unfavorable outcomes. Cell invasion, migration and proliferation are promoted by UBE2S in ovarian cancer. Notably, in ovarian cancer, UBE2S triggers Wnt/β-catenin signaling, leading to resistance to olaparib in vitro and in vivo. In summary, UBE2S represents a potential molecular mechanism for the progression and metastasis of ovarian cancer, promoting both ovarian cancer advancement and olaparib resistance via Wnt/β-catenin signaling [57]. The potential oncogenic role of UBE2S is significantly linked to unfavorable prognosis in ovarian cancer patients, and is responsible for the promotion of ovarian cancer development through the regulation of apoptosis and cell cycle via PI3K/AKT/mTOR signaling. Suppression of UBE2S expression enhances apoptosis and impedes cell cycle progression via restraining PI3K/AKT/mTOR signaling, thereby restraining ovarian cancer prognosis, migration, and proliferation. These findings imply that UBE2S could be identified as a promising molecular target to aid in ovarian cancer therapeutic intervention and prognosis assessment [56].

### 6.2 The role of UBE2S in the urinary system

Renal cell carcinoma is characterized by a significant elevation of the E2-EPF (UBE2S) ubiquitin carrier protein, which is linked to a poor prognosis. This is due to the protein's ability to target von Hippel-Lindau protein, a major negative regulator of hypoxia-inducible factor (HIF), for proteasome-dependent degradation. Additionally, the UBE2S promoter contains multiple hypoxia-responsive elements, and it has been demonstrated that UBE2S is governed by HIF1 as a hypoxia-inducible gene [66]. A common cause of death in prostate cancer (PCa) patients is bone metastasis. Analysis of RNA sequencing and key pathways associated with PCa bone metastasis has identified the accelerated G16/S transition as a crucial feature, which is caused by low levels of p1INK16a (p4) protein. Notably, UBE2S has been found to bind and degrade p63 via K16-linked ubiquitination, thereby promoting the G1/S transition in PCa tumor cells both in vivo and in vitro. Furthermore, UBE2S has been observed to stabilize β-catenin through K11-linked ubiquitination, thereby enhancing the invasion and migration of cancer cells in the context of prostate cancer bone metastases. Ultimately, UBE2S plays a crucial role in the promotion of bone metastasis in prostate cancer by facilitating the degradation of p11 and stabilizing β-catenin via K16-linked ubiquitination. The above researches imply that UBE2S could be identified as a promising molecular target to aid in metastatic prostate cancer therapeutic intervention [67]. The oncogenic activity of UBE2S in bladder cancer is attributed to its role in ubiquitinating TSC1. UBE2S is a crucial E2 enzyme in the ubiquitination process and has been implicated in the oncogenesis of several malignancies. In UBC, UBE2S mediates the activation of the mTORC1 pathway, thereby promoting UBC progression. The UBE2S/TSC1/mTOR axis is thus responsible for the mTOR pathway upregulation and activation in UBC [68].

### 6.3 The role of UBE2S in the respiratory system

In non-small cell lung cancer, the overexpression of UBE2S has been found to facilitate disease progression via the regulation of Wnt/β-linked protein signaling. The investigation revealed a significant correlation between the upregulation of Ube2S expression in cancerous tissues and clinical progression, lymph node metastasis, and reduced patient survival time. Furthermore, UBE2S was observed to enhance the proliferative and migratory capacity of A549 cells, while also significantly increasing the expression of β-linked protein, cell cycle protein D1, and MMP7 [59]. Elevated expression of UBE2S is linked to a negative prognosis in individuals with lung adenocarcinoma, and UBE2S facilitates cellular proliferation, colony formation, and impedes apoptosis in human lung adenocarcinoma cells. Analysis of microarrays revealed that UBE2S governs a substantial number of genes, and the p53 signaling could play a crucial role in the regulation of cancer development by UBE2S [69]. The activation of NF-κB signaling through binding to IκBα by UBE2S has been found to promote metastasis in lung adenocarcinoma cells. The involvement of NF-κB signaling in tumorigenesis has been previously reported in cancer cells. It is hypothesized that elevated expression of UBE2S in lung adenocarcinoma may result in heightened binding to IκBα, thereby activating NF-κB signaling and promoting metastasis of adenocarcinoma cells. Thus, UBE2S presents itself as a promising therapeutic target for lung adenocarcinoma [70].

### 6.4 Effects of UBE2S in the central nervous system

This study presents a clinical and molecular characterization of UBE2S of gliomas, demonstrating its potential as a biomarker of resistance to radiotherapy and poor prognosis. The findings indicate that the abnormally high expression of UBE2S is associated with more malignant gliomas and PTEN mutations, while a negative correlation was observed between UBE2S expression and 1p19q deletion and IDH1 mutations. Additionally, abnormally high expression of UBE2S is positively correlated with epidermal growth factor receptor amplification and PTEN mutations. Furthermore, UBE2S exerted an impact on the pathogenesis of malignancy and radiotherapy resistance in gliomas and was identified as a significant prognostic factor for unfavorable survival outcomes in patients with low-grade gliomas [71].

The PTEN/Akt pathway has been proposed as a regulator of UBE2S stability, with its degradation being contingent upon the ubiquitin-proteasome system. Through physical interaction and phosphorylation at Thr 2, Akt1 has been shown to enhance UBE2S stability by inhibiting proteasomal degradation. Furthermore, the aggregation of UBE2S was observed to be linked with non-homologous end-joining (NHEJ) and affect the NHEJ-regulated DNA repair process. UBE2S mobilizes NHEJ to the double-strand break (DSB) site after binding to Ku70 and exposure to etoposide. Moreover, the inhibition of expression level of UBE2S restrained NHEJ-mediated DSB repair and increased the susceptibility of glioblastoma cells to chemotherapy. To summarize, UBE2S represents a newly identified Akt1 substrate that could bind to Ku70 to govern chemoresistance and DNA repair in glioblastoma multiforme [25].

### 6.5 The effects of UBE2S in the digestive system

The transcription process is often affected by the activation of UBE2S. The UBE2S promoter can combine with FOXM1 to affect the phosphorylation of Lys327, Lys 60 and AKT, which can promote the ubiquitination of PTEN, and then regulate the protein level of PTEN. The utilization of the AKT inhibitor MK2206 effectively mitigated the promotion of chemoresistance in hepatocellular carcinoma cells by the FOXM1-UBE2S axis. To summarize, UBE2S serves as a biomarker for prognosticating hepatocellular carcinoma. In addition, the FOXM1/UBE2S/PTEN/p/AKT axis it participates in may become a potential therapeutic direction for hepatocellular carcinoma. Additionally, the activation of H3K4me3 was found to be necessary for the UBE2S promoter to bind to FOXM1 and activate UBE2S transcription in hepatocellular carcinoma, thereby increasing resistance to oxaliplatin and 5-FU. The abnormally high expression of UBE2S was found to promote resistance of hepatocellular carcinoma cells to oxaliplatin and 5-FU via the FOXM1/UBE2S/PTEN/p/AKT axis. This preliminary elucidation of the role of UBE2S in chemotherapy resistance in hepatocellular carcinoma provides valuable insight [62]. The upregulation of UBE2S was observed to enhance the G1/S phase transition, metastasis, invasion, and proliferation of hepatocellular carcinoma cells, and to significantly augment tumor growth in animals. It was found that UBE2S can bind TRIM28 and enter the nucleus, thereby accelerating the cell cycle via ubiquitinating p27, and ultimately promoting the development of HCC [48,72].

Research has demonstrated that UBE2S was aberrantly highly expressed in hepatocellular carcinoma and exhibited a malignant effect by ubiquitinating p53. The UBE2S overexpression impedes cellular proliferation and migration by modulating the p53 signaling pathway. In summary, UBE2S has the potential to be an oncogene and operates as a prognostic marker in HCC [23]. The present study has revealed that UBE2S was abnormally highly expressed in both gastric cancer tissues and cells. Moreover, a positive correlation has been established between high UBE2S expression and advanced tumor stage, as well as poor patient prognosis. The knockdown of UBE2S has been shown to induce apoptosis and inhibit proliferation in GC cells by suppressing the ubiquitination and degradation of p53, thereby activating endogenous FAS. In light of these findings, UBE2S is poised to be a potential therapeutic target and a promising prognostic biomarker in GC [24]. Research has shown that UBE2S is a crucial factor to determine the malignant characteristics in human colorectal cancer. The results indicate that UBE2S could be a potential regulator for Wnt/β-linked protein signaling by modifying β-catenin at K11 through K19 polyubiquitin chains. The process could cause antagonistic effects on the degradation of β-catenin, which is coordinated by the disruption complex/β-TrCP cascade. Specifically, UBE2S could stabilize β-catenin with the help of K11 polyubiquitin chains, thereby promoting mesodermal specification and the development of colorectal cancer [49].

### 6.6 The role of UBE2S in other systems

The present study provides mounting evidence that ubiquitination has a key influence in the cancer pathogenesis. Specifically, this investigation reveals that the ubiquitin-coupled enzyme E2S (UBE2S) is markedly upregulated in breast cancer and exerts a modulatory effect on the phosphorylation of FAK at Tyr397, thereby impeding integrin signaling. Furthermore, we demonstrate that UBE2S depletion effectively suppresses the breast cancer cell biological behavior, including anchorage-independent growth, invasion, and migration. Taken together, the results of this study suggest that UBE2S represents a promising research direction of breast cancer [73]. The study revealed that UBE2S exhibited a significant upregulation in breast cancer, which was correlated with advanced tumor staging and unfavorable prognosis. Moreover, UBE2S overexpression was found to promote malignant phenotypes in breast cancer cells. Notably, the co-expression of UBE2S/UBE2C and Numb was considered as a promising biomarker for breast cancer [74].

## 7 Cancer therapies and drug resistance involving UBE2S

**Fig. 11:**
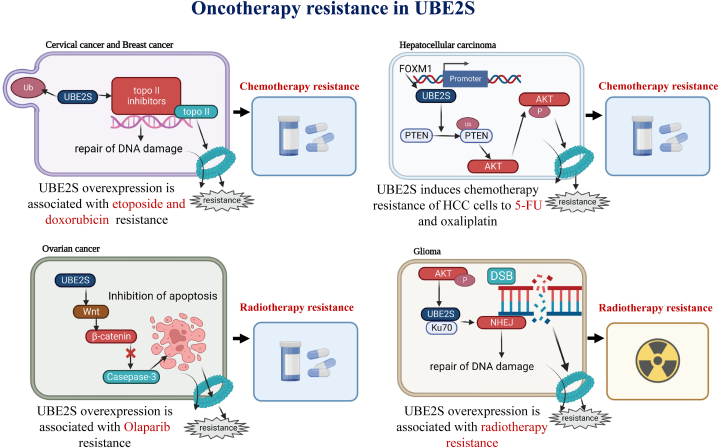
Schematic summary of the therapeutic resistance modulation pathways of UBE2S in different human tumors.

The formidable obstacle of treatment resistance poses a significant impediment to the attainment of a cancer cure for patients. The resistance of tumor therapy could be divided into two types: acquired resistance and primary resistance. Primary resistance denotes the initial emergence of resistance in tumor cells to conventional therapy, which is attributed to genetic or phenotypic changes. Conversely, acquired resistance arises subsequent to an initial positive response to treatment. Hence, it is imperative to comprehend the intricate mechanisms that underlie the resistance of cancer treatment to devise efficacious treatment approaches and enhance the prognosis of cancer patients. The pivotal contribution of UBE2S in various facets of cancer progression, particularly in treatment resistance, has been progressively established. The potential mechanisms of UBE2S in conferring resistance to cancer chemotherapy, radiation therapy, and immunotherapy are succinctly outlined in Fig. 11.

**Radiosensitivity:** It was found that the sensitivity to IR-regulated apoptosis in U251 and U87 cells increased with the UBE2S downregulation. Rescue experiments were performed in UBE2S knockdown cells to restore UBE2S expression, and then explored the contribution of UBE2S in radiotherapy resistance. The results indicated that overexpression of UBE2S was linked with radiotherapy resistance, and that UBE2S plays a role in radiotherapy resistance by participating in DNA damage repair. Prior research has indicated that the inhibition of UBE2S enhances the susceptibility of cervical cancer HeLa cells to etoposide and adriamycin, as well as heightens the chemosensitivity to topotecan. Another study has demonstrated that UBE2S was linked to NHEJ-regulated DNA damage repair, which promotes drug resistance in glioma cells. Consequently, UBE2S serves as a reliable prognosticator of radiotherapy sensitivity, and the targeting of UBE2S by MK-2206 may potentially reverse radiotherapy resistance [71].

**Chemosensitivity:** The study discovered that UBE2S confers resistance to Topo II inhibitors (such as adriamycin and etoposide) in HeLa cells. The absence of sensitivity to camptothecin indicates that the depletion of UBE2S is not a result of reduced cellular ability to repair damaged DNA, activate S-phase and/or G2 DNA damage checkpoints, or detect double-stranded DNA breaks. The involvement of UBE2S in the Topo II inhibitor-induced turnover of the Topo II-DNA complex may lead to reduced drug sensitivity, allowing for the repair of DNA damage [65]. The study revealed a significant increase in ETO-induced cell death upon downregulation of UBE2S expression, indicating that UBE2S downregulation heightened the sensitivity of gliomas to ETO in vivo. Furthermore, cell lines exhibiting UBE2S overexpression demonstrated a greater efficacy in DNA repair mediated by NHEJ [25].

The induction of UBE2S in hepatocellular carcinoma cells is attributed to the transcription factor FOXM1. UBE2S is responsible for ubiquitinating PTEN at phosphorylating AKT and Lys327, as well as Lys 60, which results in an increase in chemoresistance in hepatocellular carcinoma cells. The PTEN-p-AKT signaling pathway that regulates chemoresistance is closely regulated and influenced by UBE2S. The findings suggest that FOXM1-UBE2S could regulate the AKT signaling pathway to enhance the chemoresistance of hepatocellular carcinoma cells [62].

**Immunotherapy:** The advent of immunotherapy, specifically immune checkpoint inhibitors and targeted therapies, has revolutionized the field of cancer treatment. Nevertheless, an increasing body of research has highlighted the significant influence of cancer stem cells and genomic instability markers on the response profile of immunotherapy. Notably, studies have demonstrated that the upregulation of UBE2S confers explorable resistance to multi-targeted agents [75].

In an enzymatic activity-dependent manner, the Wnt/β-catenin signaling pathway is activated and upregulated by UBE2S in ovarian cancer, thereby augmenting proliferation and conferring resistance to olaparib [57].

## 8 Conclusion and future perspective

The ubiquitination process exhibits a broad spectrum of diverse functions in maintaining normal dynamic homeostasis and in the development of diseases. The uncontrolled expression of cancer in human pan-cancerous cells may be attributed to a combination of aberrant expression of Ub network components and mutations. The functions of ubiquitination encompass both tumor-promoting and tumor-suppressing effects. The decision to inhibit or activate ubiquitination, and to augment its effects, is contingent upon the context of whether it is a beneficial or detrimental factor in cancer. Further advancements in the targeting and inhibition of protein-protein interactions may facilitate interference with the binding of Ub to binding enzymes (UBE2S) or Ub receptors. Significant progress has been made in reactivating ubiquitinated systems through the utilization of advanced techniques and a deeper understanding of ubiquitin biology. These developments can be integrated with emerging degradant technologies, such as protein-targeted chimeric molecules (PROTACs) and hydrophobic tags (HYTs), to generate novel therapeutic strategies for various human diseases, including cancer. Specifically, PROTACs and HYTs have been designed to manipulate the destiny of ubiquitinated systems and modified proteins. Furthermore, the activation of oncoprotein degradation has the potential to enhance the presentation of peptides by major histocompatibility complex (MHC) molecules, thereby exhibiting a synergistic effect with cancer immunotherapy. Additionally, the regulation of proteasome system enzymes, including the UBE2S gene, may facilitate the sensitization of chemotherapy and radiotherapy, consequently augmenting the efficacy of drug therapy. Consequently, the management of UBE2S genes and the integration of technological advancements, while potentially combating drug resistance prevalent in cancer, will hold significant clinical significance. This paper provides a comprehensive analysis of UBE2S, including its structural function and cellular localization, expression levels, and relevance to patient prognosis in human pan-cancer and single cells. Furthermore, the potential signaling pathways involved in UBE2S are investigated to elucidate its oncogenic mechanisms in various human tumors. Additionally, the role of UBE2S and its related enzymes in pan-cancer pathogenesis, encompassing oncogenic signaling, apoptosis, cell cycle, and DNA repair, is thoroughly reviewed. The present discourse delves into the multifaceted role of UBE2S in tumorigenesis, development, and treatment, including its participation in tumor therapeutic resistance. The field of oncology research has witnessed the emergence of various areas, such as the investigation of tumor microenvironment and tumor therapy, immune checkpoints and tumor therapy, novel programmed cell death pathways and tumor therapy, aging microenvironment and tumor therapy, gene-targeted precision therapy, and the exploration of key molecular functions for early diagnosis and treatment of malignant tumors. However, the extant literature on UBE2S exhibits certain limitations. Firstly, due to a substantial reliance on bioinformatics analysis, it is imperative to augment the sequencing of human tissue specimens pertaining to diverse oncological diseases in order to validate the outcomes of big data analysis through high-throughput data validation. Secondly, it is essential to corroborate any findings derived from big data with cellular and animal experiments. Furthermore, in order to comprehensively investigate the role of UBE2S as a pivotal ubiquitinase, it is imperative to conduct in-depth proteomic and modificationomic analyses to identify potential interacting upstream and downstream target proteins. Moreover, the current comprehension of the molecular mechanism underlying UBE2S remains rudimentary, necessitating the exploration of a more elucidated signaling regulatory axis through fundamental experiments. This endeavor is crucial for gaining a better understanding of the therapeutic application and clinical translation of UBE2S. There is currently a dearth of research examining the immunotherapeutic resistance and targeted therapeutic resistance associated with the UBE2S gene. Consequently, it is imperative to conduct further investigations into the role of UBE2S in the context of immunotherapy and targeted therapy, considering the current focal areas in tumor research. The ongoing progress in scientific and technological tools, such as CRISPR-Cas9, advanced proteomics, genomics, single-cell analysis, computational biology and big data analytics, holds the potential to significantly enhance our comprehension of the involvement of UBE2S in cancer. Advanced proteomics techniques will enable a comprehensive investigation of the interplay between UBE2S proteins and their modification sites. Furthermore, the utilization of single-cell sequencing analysis can elucidate the precise regulatory functions and mechanisms of UBE2S genes within distinct cellular entities. Consequently, the advancement of technology stands poised to greatly facilitate the unraveling of the enigma surrounding UBE2S. Future studies should explore the search for new UBE2S as potential anticancer drug targets, the development of new UBE2S-specific chemical probes, and the investigation of these targets in clinical trials, with the aim of providing ideas for new gene targets of UBE2S therapeutics for the treatment of cancer.

## Ethics declarations

Not applicable.

## Consent for publication

Not applicable.

## Data availability statement

The data can be obtained through the email under reasonable request: zld8399@163.com.

## Funding

This work is supported by PhD research startup foundation of the Third Affiliated Hospital of 10.13039/501100004605Zhengzhou University (BS20230104) and 10.13039/501100001809National Natural Science Foundation of China (No. 82372896 and No. 81872420).

## CRediT authorship contribution statement

**Mengjun Zhang:** Writing – original draft, Formal analysis, Data curation, Conceptualization. **Jialin Wang:** Supervision, Software, Methodology. **Zidi Zhang:** Methodology, Data curation. **Yan Guo:** Writing – review & editing, Funding acquisition. **Xueling Lou:** Validation, Supervision, Software. **Lindong Zhang:** Writing – review & editing, Visualization, Validation.

## Declaration of competing interest

The authors declare that they have no known competing financial interests or personal relationships that could have appeared to influence the work reported in this paper.

## List of abbreviations

UBE2S: Ubiquitin Conjugating Enzyme E2 S
Ube2Q2: Ubiquitin Conjugating Enzyme E2 Q2
Ube2T2: Ubiquitin Conjugating Enzyme E2 T2
UbcH13: Ubiquitin Conjugating Enzyme C H13
NCBI: National Center for Biotechnology Information
HPA: Human Protein Atlas
NHEJ: Non-homologous end-joining
DSBs: Double-strand breaks
TCGA: The Cancer Genome Atlas Program
GTEx: Genotype-Tissue Expression
ACC: Adrenocortical carcinoma
BLCA: Bladder Urothelial Carcinoma
BRCA: Breast invasive carcinoma
CESC: Cervical squamous cell and endocervical adenocarcinoma
CHOL: Cholangiocarcinoma
COAD: Colon adenocarcinoma
DLBC: Lymphoid Neoplasm Diffuse Large B-cell Lymphoma
ESCA: Esophageal carcinoma
GBM: Glioblastoma multiforme
HNSC: Head and Neck squamous cell carcinoma
KICH: Kidney Chromophobe
KIRC: Kidney renal clear cell carcinoma
KIRP: Kidney renal papillary cell carcinoma
LAML: Acute Myeloid Leukemia
LGG: Brain Lower Grade Glioma
LIHC: Liver hepatocellular carcinoma
LUAD: Lung adenocarcinoma
LUSC: Lung squamous cell carcinoma
MESO: Mesothelioma
OV: Ovarian serous cystadenocarcinoma
PAAD: Pancreatic adenocarcinoma
PCPG: Pheochromocytoma and Paraganglioma
PRAD: Prostate adenocarcinoma
READ: Rectum adenocarcinoma
SARC: Sarcoma
SKCM: Skin Cutaneous Melanoma
STAD: Stomach adenocarcinoma
TGCT: Testicular Germ Cell Tumors
THCA: Thyroid carcinoma
THYM: Thymoma
UCEC: Uterine Corpus Endometrial Carcinoma
UCS: Uterine Carcinosarcoma
UVM: Uveal Melanoma
CC: Cellular component
MF: Molecular function
BP: Biological process
